# Mandarin Juice Powder Production by Foam Mat Drying: Optimization and Determination of Drying Kinetics

**DOI:** 10.1021/acsomega.6c00329

**Published:** 2026-05-20

**Authors:** Sevgi Şule ÖZTÜRK, Ahsen RAYMAN ERGÜN, Taner BAYSAL

**Affiliations:** 37509 Ege University, Engineering Faculty, Food Engineering Department, 35100 Bornova, Izmir, Turkey

## Abstract

Foam mat drying is a promising development with the advantages of low cost and can be used as an alternative to drum and spray drying for drying aqueous foods. The economic value and importance of mandarin fruits are increasing day by day due to their rich nutritional content, ease of marketing, easy peeling compared to other citrus fruits, the existence of varieties that ripen at different times, and their resistance to cold. The aim of this study was to optimize the production conditions of mandarin juice foam with response surface methodology, to examine the drying kinetics of hot air and freeze-drying methods, and to express the drying behavior of mandarin juice foams with mathematical modeling. Egg albumen was used as a foaming agent at different concentrations (0, 1.5, and 3% w/w), and carboxymethyl cellulose (0, 1, and 2% w/w) was used as a foam stabilizer. Optimum production conditions were found as a whipping time of 6 min 20 s, an egg albumen concentration of 3% (w/w), and a carboxymethyl cellulose concentration of 2% (w/w). Under the optimized conditions, density, stability, and expansion values of foam were found as 0.262 (g/cm^3^), 348.955, and 302.516%, respectively. The drying times for temperatures of 50, 60, 70, and −52 °C were determined as 105, 82, 70, and 180 min, respectively. Based on mathematical modeling, the “Page” model was found to be suitable for all temperature values.

## Graphical abstract

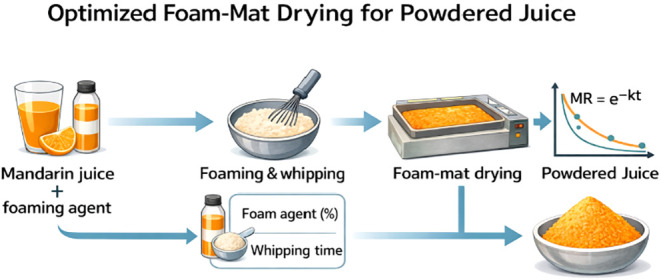

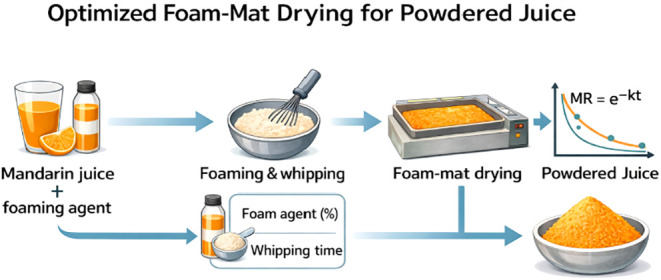

## Introduction

The foam drying method is an innovative drying method defined as the drying of foam obtained by mixing liquid or semisolid foods with a foam stabilizer and/or foaming agent and spreading it as a thin layer.^1–4^ The foam drying method is an economically efficient process due to its simplicity and shorter drying process time compared to other drying methods.^5^ It is possible to dry heat-sensitive, sticky, viscous, and difficult-to-dry foods such as puree and water forms of fruits and vegetables, milk, soluble coffee, and jam.^6,7^

In the literature, many fruits were foam-dried such as banana,^8–15^ apple/apple juice,^16–21^ lime,^22–24^ dragon fruit,^25–27^ mango,^28–33^ pineapple,^34–36^ and papaya,^6,37–41^ and there are also studies with many vegetables such as tomatoes/tomato juice,^42–49^ potato,^50–53^ onion,^54^ and garlic.^55^

In foam mat drying, the selection of foaming and stabilizing agents and their concentrations, as well as varying whipping times in foam formation, is very crucial for the quality of powder products. Compounds that increase foam stability are called foam stabilizers.^56^ In previous research, carboxymethyl and methyl cellulose (CMC), which have a polysaccharide structure, are used as foam stabilizers.^57,56,58^ It was seen from the literature that there is a lack in the optimization of the foam mat drying method and drying kinetics. Only limited studies^59^ aimed at examining the effect of foaming agent concentration on the drying of jackfruit pulp by using the response surface methodology. Also, there is a lack in research studies about the foam mat drying of citrus fruits such as orange,^60^ bitter orange,^61^ and mandarin^62^ that were studied previously, but they investigated the quality only and/or used microwave technology.

Mandarin attracts attention among citrus fruits thanks to secondary metabolites. The unique characteristic properties of the mandarin fruit are its color, unique flavor elements, and essential oils that are valuable for health.^63,64^ Flavonoids with polyphenolic structures are called secondary metabolites of plants.^65^ It is reported that citrus fruits are rich in flavonoids.^66^ Hesperidin is the dominant flavonoid type in mandarin fruit,^67^ exhibits vitamin-like activity, maintains vascular health,^68^ and provides anticarcinogenic effects on stomach cancer, colon cancer, and lung cancer.^69^ On the other hand, there has been a 12% increase in the level of mandarin production. In addition, mandarin fruit production in the world has increased by 37% in the last 10 years. China, known as the homeland of citrus fruits, meets 72% of the world’s mandarin production. Turkey is the third most mandarin-producing country in the world, with 1.9 million tons (5%) of mandarin production.^70,71^

This investigation, unlike the literature, aimed at optimizing the agent/stabilizer concentration and whipping time in obtaining mandarin juice foam by the foam drying method with two different methods (hot air and freeze-drying) to examine the drying characteristics of drying processes and to determine the appropriate model for the study by mathematical modeling.

## Materials and Methods

### Materials

The mandarin fruits used in the study were purchased from Buca Fruit and Vegetable Market (İzmir, Turkey) and washed with tap water. Then, they were peeled and squeezed into mash form using a juicer (Moulinex, JU5000, Turkey). The obtained fruit mash was filtered to remove the particle residues. The mandarin juice was filled in 200 mL bottles and stored in a freezer (−25 °C) (Uğur, UCF 310 SSL). Egg albumen powder was used as a foaming agent, and food-grade carboxymethyl cellulose (E466) (Alfasol, Istanbul, Turkey) was used as a foam stabilizer.

## Methods

### Drying Techniques

For drying the samples with hot air, a tray dryer (Eksis TK, Turkey) was used. After the mandarin juice foams were spread in glass Petri dishes with a thickness of 3 mm, they were subjected to the drying process in the tray dryer at 50, 60, and 70 °C temperatures and a 1 m/s air flow rate. In order to obtain drying kinetic data related to the hot air drying technique, the Petri dishes were weighed at 5 min intervals, and the drying process was continued until there was no difference between the last two weighings. At the end of the period, the moisture content of the mandarin powder samples was aimed to reach the equilibrium moisture content.

For freeze-drying the mandarin foam samples, a lyophilizer (Christ α 1–4 Ldplus, Germany) was used. Before the freeze-drying process, the samples were spread in glass Petri dishes with 3 mm thickness, covered with filter paper and a stretch film, and placed in a shock deep freezer (MDF-U443, Sanyo, Osaka, Japan) at −40 °C for 24 h. Temperature and pressure parameters for drying of the samples were determined as −52 °C and 0.001 mbar, respectively. Weight measurements were taken at 30 min intervals, and the drying process was continued until no difference was observed between the last two measurements.

### Experimental Design and Optimization

The optimization of the process parameters to be selected for the production of mandarin juice powder by the foam drying method was carried out using face-centered central composite design in response surface methodology (RSM) by using the Design Expert v.7.0 (Minneapolis) package program. The levels of the independent variables for the optimization of the foam formation process with carboxymethyl cellulose are shown in Table 1, and the experimental design is shown in Table 2.

**Table 1:** Independent Variable Levels in Experimental Design for RSM

	coded levels		
independent variables	–1	0	1
egg albumen	0.00	1.50	3.00
whipping time	3.00	5.00	7.00
carboxymethyl cellulose (CMC)	0.00	1.00	2.00

**Table 2:** Experimental Design Used in the Optimization for Foam Formation

	independent variables		
experiments	egg albumen (%w/w)	carboxymethyl cellulose (CMC) (%w/w)	whipping time (min)
1	0.00	0.00	3.00
2	0.00	0.00	7.00
3	3.00	0.00	3.00
4	3.00	0.00	7.00
5	0.00	2.00	3.00
6	0.00	2.00	7.00
7	3.00	2.00	3.00
8	3.00	2.00	7.00
9	1.50	1.00	3.00
10	1.50	1.00	7.00
11	0.00	1.00	5.00
12	3.00	1.00	5.00
13	1.50	0.00	5.00
14	1.50	2.00	5.00
15	1.50	1.00	5.00
16	1.50	1.00	5.00
17	1.50	1.00	5.00

Foam density (FD), foam stability (FS), and foam expansion (FE) values of mandarin juice foams obtained by applying the trial plans were selected as the response. The significant terms in the model obtained for the response were determined by variance analysis (ANOVA). The optimum point was determined under the operating conditions where the foam density value was minimum, and the foam stability and foam expansion values were maximum. The desirability function approach was used for the optimization. In order to verify the optimum point, three repeated trials were performed at the solution point given by the program, and the experimental values and the values estimated from the model were compared. The residual standard error (RSE) percentages were calculated for each response. Whether there was a statistically significant difference between the results of the verification analysis and the results estimated from the model was determined by applying the single-sample *t*-test in the SPSS package program.

### Foam Characteristics

#### Foam Density

The foam density value of mandarin juice foams was calculated by dividing the foam weight by the foam volume according to the method given in ref^28^ (eq 1).

1 $$\mathrm{Foam density}\left(g/{\mathrm{cm}}^{3}\right)=\frac{\mathrm{weight of foam}\left(g\right)}{\mathrm{volume of foam}\left({\mathrm{cm}}^{3}\right)}$$

##### Foam Stability

The foam stability value was calculated by modifying the method specified in ref.^72^ The produced mandarin juice foams were kept at room temperature for 3 h, and the volume was measured at the end of the period. The foam volume at the end of 3 h is shown as Vt, and the initial foam volume is shown as Vi (eq 2).

2 $$\mathrm{Foam stability}\left(\%\right)=\frac{\mathrm{foam volume at the end of the time}\left({\mathrm{cm}}^{3}\right)}{\mathrm{initial volume of foam}\left({\mathrm{cm}}^{3}\right)}\times 100$$

##### Foam Expansion Value

The foam expansion value is defined as a measure of the amount of air trapped within the structure by the foam.^73^ It was calculated by dividing the volumes of the foams obtained with mandarin juice before and after the foam was formed (eq 3).^74,73^ The initial mandarin juice volume was shown as V1, and the volume after the foam was shown as V2.

3 $$\mathrm{Foam expansion}\left(\%\right)=\frac{V_{2}\left({\mathrm{cm}}^{3}\right)-V_{1}\left({\mathrm{cm}}^{3}\right)}{V_{1}\left({\mathrm{cm}}^{3}\right)}\times 100$$

### Drying Characteristics

The drying rate (*R*) value was calculated for each period according to eq 4.^75^ Drying surface area (m^2^) is represented by letter *A* in the equation.

4 $$R=-\left(\frac{w_{s}}{A}\right)\;\times \;\left(\frac{d_{x}}{d_{t}}\right)$$

The moisture ratio (MR) value in the models is calculated based on eq 5.^76^ *M*_i_ in the equation represents the initial moisture content, *M*_e_ represents the equilibrium moisture content, and *M_t_* represents the moisture content at time *t*.

5 $$\mathrm{MR}=\frac{M_{t}-M_{e}}{M_{0}-M_{e}}$$

### Mathematical Modeling

In order to express the time-dependent changes in the drying kinetic values obtained experimentally with mathematical models, seven different drying models in Table 3 were used.^77^

**Table 3:** Mathematical Models Used in the Research

model	model equation	refs
Lewis (Newton)	MR = exp(−*kt*)	^95^
Page	MR = exp(−*kt^n^*)	^96^
Henderson and Pabis	MR = *a*exp(−*kt*)	^97^
logarithmic	MR = *a*exp(−*kt*) + *c*	^98^
two-term	MR = *a*exp(−*kt*) + *b*exp(−*ct*)	^99^
exponential two-term	MR = *a*exp(−kt) + (1–*a*)exp(−*kat*)	^100^
diffusion approach	MR = *a*exp(−*kt*) + (1–*a*)exp(−*kbt*)	^101^

In order to evaluate the compatibility of the mathematical models used with the experimental data obtained from drying mandarin juice foam, nonlinear regression analysis was performed using the SPSS package program. The linear regression coefficient (*R* ^2^) was calculated with the root-mean-square error (RMS) and chi-square (χ2) values. RMS and χ2 values were calculated according to eqs 6 and 7.^78^ MR_exp,*i*_ in the mathematical expression represents the experimental moisture rate, MR_pre,*i*_ represents the moisture rate estimated from the model, *N* represents the number of observed experimental data, and *n* represents the number of coefficients in the model.

6 $$\mathrm{HKOK}=\sqrt{\frac{\sum_{i=1}^{n}{\left({\mathrm{MR}}_{\mathrm{pre},i}-{\mathrm{MR}}_{\exp,i}\right)}^{2}}{N}}$$

7 $$\chi^{2}=\frac{\sum_{i=1}^{n}{\left({\mathrm{MR}}_{\exp,i}-{\mathrm{MR}}_{\mathrm{pre},i}\right)}^{2}}{N-n}$$

#### Statistical Analysis

In order to evaluate the analysis results, the SPSS (IBM SPSS, 2011, 20.0 for Windows Version; SPSS Inc., Chicago, Ill.) software program was used. Results were evaluated at a *p* < 0.05 significance level with one-way ANOVA, Duncan multiple comparison, and nonlinear regression methods. The optimization of the process parameters to be selected for the production of mandarin juice powder by the foam drying method was carried out using face-centered central composite design in response surface methodology (RSM) by using the Design Expert v.7.0 (Minneapolis) package program. Foam density (FD), foam stability (FS), and foam expansion (FE) values of mandarin juice foams obtained by applying the trial plans were selected as the response. The significant terms in the model obtained for the response were determined by variance analysis (ANOVA).

## Results and Discussion

### Optimization of Foam Production Conditions with Response Surface Methodology

#### Foam Density

The response surface analysis (Figure 1) indicates that the foam density is strongly influenced by egg albumen concentration and whipping time. Increasing egg albumen leads to a decrease in foam density due to the enhanced formation of protein networks that trap air effectively. However, excessive protein concentrations can increase viscosity, limiting air incorporation and slightly raising density. Whipping time exhibits a similar trend: short whipping yields weak protein networks and higher density, while moderate whipping allows optimal air entrapment and lowers the density. Overwhipping can induce protein overcoagulation, resulting in bubble collapse and increased density.

**Figure 1:**
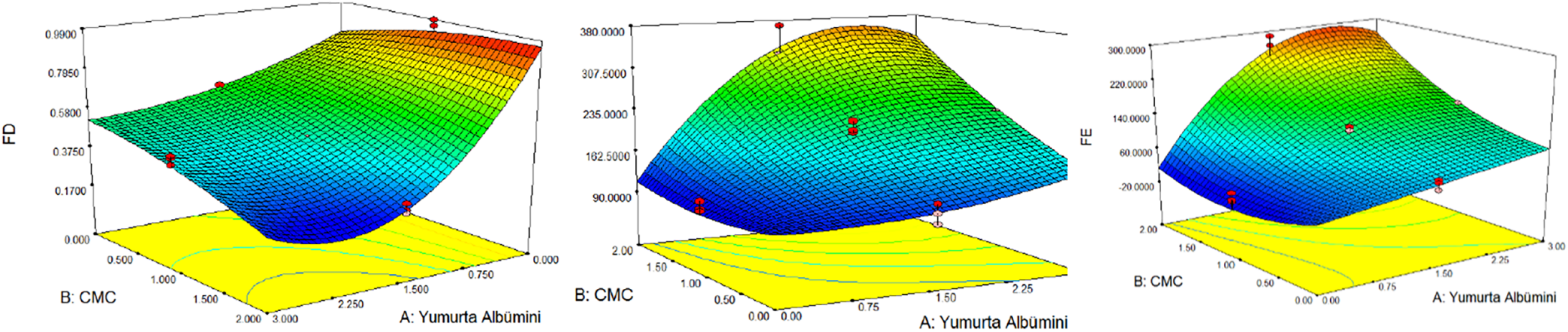
Response surface plot for foam density, stability, and expansion value.

The CMC concentration exhibits a dual role. Moderate CMC increases continuous-phase viscosity, stabilizing bubbles and reducing foam density, whereas higher CMC levels create an overly rigid network, restricting air incorporation and increasing density. Significant interactions were observed between egg albumen and whipping time, indicating that the effect of whipping is protein-dependent: high albumen with moderate whipping achieves the lowest density, while overwhipping at high protein levels increases density. Mechanistically, the interplay among protein unfolding, viscoelastic film formation, and CMC-mediated viscosity governs the foam structure. These trends are consistent with previous studies on citrus foams, where acidic pH promotes protein unfolding, while neutral pH fruits such as banana or mango typically show higher foam density due to less efficient protein stabilization.^79^ It was determined that the foam density (FD) value used as the response parameter in the optimization study varied between 0.237 and 0.991 g/cm^3^.^61^ They used citrus fruit as a raw material for microwave-assisted foam drying hybrid system components. This study investigated the drying and color kinetics, powder product structure, and antioxidant activity behavior of samples subjected to intermittent drying at different power values (90, 180, and 360 W). The foam density, foam stability, and foam expansion values of egg albumin with different components (40, 50, and 60% (w/w)) were investigated. The analyses showed that the foam density ranged from 0.899 to 0.948 g/cm3, and the foam expansion varied between 9% and 16%.^61^

The optimum foam density value for the foam drying process was found to be between 0.2 and 0.5 g/cm^3^ for tomato powder by ref.^79^ The ANOVA table given by the program for the foam density (FD) value is listed as Table 4.

**Table 4:** ANOVA Table and Model Parameters Obtained for the Foam Density Response

source	sum of squares of errors	Df	mean squared error	*F*-value	*p*-value
model	2.70	12	0.23	60.66	<0.0001^a^
A	1.84	1	1.84	496.66	<0.0001
B	0.17	1	0.17	46.12	<0.0001
C	0.043	1	0.043	11.71	0.0017
AB	0.23	1	0.23	62.20	<0.0001
AC	5.132 × 10^–3^	1	5.132 × 10^–3^	1.38	0.2482
BC	1.016 × 10^–3^	1	1.016 × 10^–3^	0.27	0.6043
A^2^	0.33	1	0.33	90.19	<0.0001
B^2^	0.012	1	0.012	3.29	0.0792
C^2^	3.225 × 10^–5^	1	3.225 × 10^–5^	8.692 × 10^–3^	0.9263
ABC	1.026 × 10^–5^	1	1.926 × 10^–5^	5.191 × 10^–3^	0.9430
A^2^B	0.096	1	0.096	25.88	<0.0001
A^2^C	0.027	1	0.027	7.29	0.0110
residue	0.012	32	3.710 × 10^–3^		
model incompatibility	7.773 × 10^–3^	2	3.887 × 10^–3^	1.05	0.3621
pure error	0.11	30	3.698 × 10^–3^		
sum	2.82	44			

a Statistically significant at the level of *p* < 0.05. A, B, and C in the table represent egg albumen, carboxymethyl cellulose, and whipping time, respectively.

The *R* ^2^ value for the foam density (g/cm^3^) was determined as 0.957. The *R* ^2^ value of the model being close to 1 is a criterion, indicating that the model is compatible with the experimental data. The difference between the adjusted *R* ^2^ (*R*_adjusted_^2^) and predicted *R* ^2^ (*R*_predicted_^2^) values being less than 0.2 is an indication that the difference is at a reasonable level. The difference between these two values was calculated to be 0.025. The adjusted *R* ^2^ and predicted *R* ^2^ values for the foam density response are shown in Table 5.

**Table 5:** *R* ^2^, Adjusted *R* ^2^, and Predicted *R* ^2^ Values of Foam Density, Stability, and Expansion

	*R* ^2^	(*R*_adjusted_^2^)	(*R*_predicted_^2^)
FD (g/cm^3^)	0.9579	0.9421	0.9171
FS (%)	0.9269	0.9025	0.8632
FE (%)		0.9145	0.8861

The second-degree equation obtained as a result of the regression analysis for the foam density (FD) response value is given in eq 8 in terms of coded variables. A, B, and C in the equation represent egg albumen, carboxymethyl cellulose, and whipping time, respectively.

8 $$\mathrm{FD}=0.47-0.25^{*}A-0.17^{*}B-0.08^{*}C-0.098^{*}\mathrm{AB}-0.02^{*}\mathrm{AC}+6.507E-003^{*}\mathrm{BC}+0.21^{*}A^{2}-0.04^{*}B^{2}+2.045E-003^{*}C^{2}+8.958E-004^{*}\mathrm{ABC}+0.14^{*}A^{2}B+0.08^{*}A^{2}C$$

The effects of agent and stabilizer concentrations on foam density (FD) values are shown in the response surface graph in Figure 1. Blue areas indicate points where the value is low, and red areas indicate points where the value is high. It is observed that as the agent and stabilizer concentration increases, the foam density value decreases; this is also in line with the previous research that as the concentration of EA increases, foaming agents migrate from the aqueous phase to air–liquid interfaces, leading to a reduction in surface tension. Consequently, this process enhances the foaming capacity while decreasing density.^61,79^

#### Foam Stability

In the optimization study, foam stability (FS) used as the response parameter varied between 100 and 400 (%). The ANOVA table given by the program for the foam stability (FS) value is listed as Table 6.

**Table 6:** ANOVA Table and Model Parameters Obtained for the Foam Stability Response of Foaming Trials

source	sum of squares of errors		mean squared error	*F*-value	*p*-value
model	3.406 × 10^5^	11	30965.74	38.03	<0.0001^a^
A	1.542 × 10^5^	1	1.542 × 10^5^	189.39	<0.0001
B	68566.11	1	68566.11	84.22	<0.0001
C	2845.75	1	2845.75	3.50	0.0704
AB	48651.42	1	48651.42	59.76	<0.0001
AC	493.57	1	493.57	0.61	0.4418
BC	29.76	1	29.76	0.037	0.8496
A^2^	15493.95	1	15493.95	19.03	0.0001
B^2^	1848.44	1	1848.44	2.27	0.1414
C^2^	208.65	1	208.65	0.26	0.6161
ABC	6.95	1	6.95	8.537 × 10^–3^	0.9269
A^2^B	18721.18	1	18721.18	22.99	<0.0001
residue	0.012	33	3.710 × 10^–3^		
model incompatibility	7.773 × 10^–3^	3	1460.85	1.95	0.1430
pure error	22484.82	30	749.49		
sum	3.675 × 10^5^	44			

a Statistically significant at the level of *p* < 0.05. A, B, and C in the table represent egg albumen, carboxymethyl cellulose, and whipping time, respectively.

The *R* ^2^ value for foam stability was determined as 0.9269. The difference between the adjusted *R* ^2^ and predicted *R* ^2^ values was determined as 0.039. (Table 5). The second-order equation obtained as a result of the regression analysis for the foam stability (FS) value in the optimization study is included in eq 9 in terms of coded variables. A, B, and C in the equation represent egg albumen, carboxymethyl cellulose, and whisking time, respectively.

9 $$\mathrm{FS}=210.09+71.69^{*}A+106.90^{*}B+9.74^{*}C+45.02^{*}\mathrm{AB}+4.53^{*}\mathrm{AC}-1.11^{*}\mathrm{BC}-44.82^{*}A^{2}+15.48^{*}B^{2}-5.20^{*}C^{2}-0.54^{*}\mathrm{ABC}-62.45^{*}A^{2}B$$

ANOVA results (Table 6) demonstrate that foam stability is predominantly influenced by egg albumen (*p* < 0.0001) and whipping time (*p* < 0.0001), with CMC alone not significant (*p* = 0.0704). Significant interactions, especially egg albumen × whipping time, indicate that stability depends on both protein content and mechanical aeration. Quadratic and higher-order interactions (e.g., A^2^B) highlight nonlinear effects. Increasing egg albumen improves stability by forming viscoelastic films that resist coalescence and drainage, while optimal whipping promotes uniform, small bubbles. Overwhipping can cause structural collapse and reduced stability. CMC, although not individually significant, enhances stability in combination with proteins by increasing viscosity, slowing drainage, and supporting the protein network.

The effects of egg albumen and carboxymethyl cellulose concentration on the foam stability (FS) value are shown in the response surface graph in Figure 1. It is observed that there is a positive correlation among egg albumen (% (w/w)) concentration, carboxymethyl cellulose (% (w/w)) concentration, and foam stability value. Similarly, it was underlined that, with an increase in the concentration of foaming agents (1–3%), the values of foam stability increased to 97.26–98.25% by using CMC in papaya pulp.^80^ As reported in ref,^37^ a reduction in pulp concentration and an increase in drainage volume lead to a decline in foam stability. In a previous research, the aim was to investigate the effect of foaming agent concentration on the drying of jackfruit pulp by foam drying and on the quality characteristics of jackfruit powder using response surface methodology. In the study, a fixed concentration of methyl cellulose (0.5% (w/w)) and different concentrations of glycerol monostearate (2, 3, and 4% (w/w)), soy protein (0.5, 1, and 1.5% (w/w)), and maltodextrin (3, 4, and 5% (w/w)) were used. Foam expansion and foam stability were evaluated. In the group containing 4% (w/w) maltodextrin + 3% (w/w) glycerol monostearate, which was determined as the optimum point, the response parameters were calculated as 69.8 and 89.4%, while in the group containing 4% (w/w) maltodextrin + 1% (w/w) soy protein, the response parameters were calculated as 74.44 and 84.80%, respectively.^79^

#### Foam Expansion

It was determined that the foam expansion (FE) value used as the third response parameter in the optimization study varied between 0 and 306.66 (%). The ANOVA table given by the program for the foam expansion (FE) value is listed as Table 7. The *R* ^2^ value of the foam expansion response parameter was determined to be 0.9145. The difference between the adjusted *R* ^2^ and predicted *R* ^2^ values obtained from the program being less than 0.2 is an indication that the difference is at a reasonable level. Table 5 shows the *R* ^2^, adjusted *R* ^2^, and predicted *R* ^2^ values of the foam expansion (FE) response.

**Table 7:** ANOVA Table and Model Parameters Obtained for the Foam Expansion Response of Foaming Experiments

source	sum of squares of errors		mean squared error	*F*-value	*p*-value
model	3.812 × 10^5^	**11**	**34652.04**	**32.10**	<0.0001*
A	1.920 × 10^5^	1	1.920 × 10^5^	177.88	<0.0001
B	59688.07	1	59688.07	55.30	<0.0001
C	2798.40	1	2798.40	2.59	0.1169
AB	53487.71	1	53487.71	49.56	<0.0001
AC	620.34	1	620.34	0.57	0.4538
BC	27.62	1	27.62	0.026	0.8739
A^2^	27753.83	1	27753.83	25.71	<0.0001
B^2^	8992.22	1	8992.22	8.33	0.0068
C^2^	453.16	1	453.16	0.42	0.5215
ABC	26.47	1	26.47	0.025	0.8765
A^2^B	16921.07	1	16921.07	15.68	0.0004
residue	35618.28	33	1079.34		
model incompatibility	7068.19	3	2356.06	2.48	0.0806
pure error	28550.09	30	951.67		
sum	4.168 × 10^5^	44			

The expansion value in the optimization study is given in eq 10 in terms of coded variables. A in the equation represents egg albumen (% (w/w)), B represents arboxymethyl cellulose (% (w/w)), and C represents whipping time (min).

10 $$\mathrm{FE}=+126.54+80.00^{*}A+99.74^{*}B+9.66^{*}C+47.21^{*}\mathrm{AB}+5.08^{*}\mathrm{AC}-1.07^{*}\mathrm{BC}+59.98^{*}A^{2}+34.14^{*}B^{2}-7.66^{*}C^{2}-1.05\mathrm{ABC}-59.37^{*}A^{2}B$$

The effects of agent and stabilizer concentrations on the foam expansion value are shown in the response surface graph in Figure 2. It is observed that there is a positive correlation between egg albumen (% (w/w)) and carboxymethyl cellulose (% (w/w)) concentrations and foam expansion value. Foam expansion, inversely related to the density, is affected by all three factors. Increasing egg albumen enhances expansion by forming stronger protein films around air bubbles, although excessive protein may limit bubble growth due to viscosity constraints. Whipping time initially promotes expansion by incorporating air and forming uniform bubbles, but overwhipping causes coalescence and reduces expansion. CMC acts as a stabilizer, enhancing foam expansion at moderate levels by slowing drainage and preventing bubble collapse; however, at higher concentrations, viscosity becomes too high, limiting air incorporation. Interactions between egg albumen and whipping time are significant: maximum expansion is observed at high protein with moderate whipping, whereas excessive whipping reduces it. Similarly, moderate CMC synergizes with protein and optimal whipping to stabilize small bubbles, whereas a high CMC impedes expansion.

**Figure 2:**
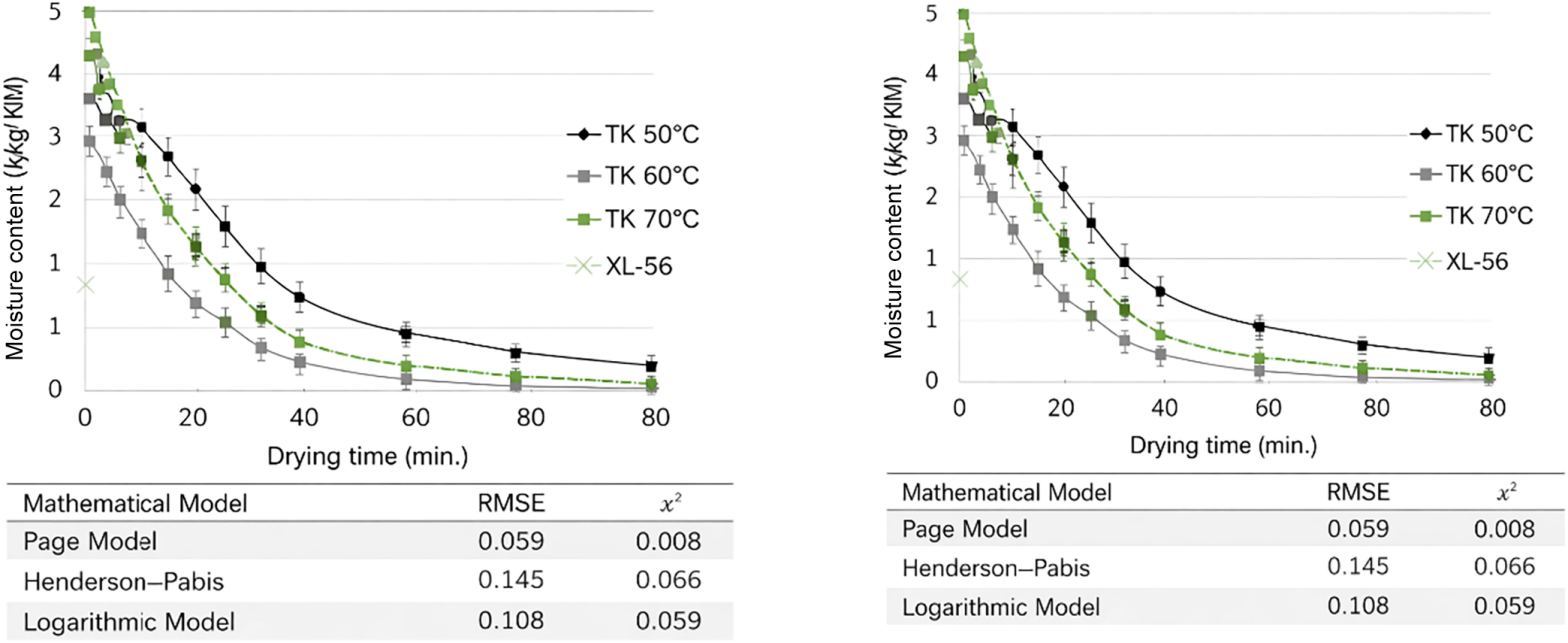
Variation of total moisture content and free moisture content of foam dried by hot air and freeze-drying methods, depending on drying time.

In contrast, with an increase in CMC concentration up to 1%, the expansion volume increases and then decreases after reaching the 1% CMC level. Additionally, CMC enhances foam stability by increasing its viscosity, which could account for the reduction in foam expansion at higher CMC concentrations.^79^ Raising the drying temperature initially caused a slight increase in the foam volume, but this was followed by a decreasing trend. This pattern is likely due to the role of the protein-based foaming agent, which is essential for forming the foam. During the whipping process, proteins undergo denaturation and arrange themselves at the air–water interface, creating a stable film that helps maintain the foam structure. However, elevated drying temperatures may compromise this stability, leading to the collapse of the foam structure and a reduction in foam expansion.^81^ Mechanistically, foam expansion is governed by the ability of proteins to lower surface tension and form viscoelastic films, while CMC enhances continuous-phase viscosity, slowing drainage, and Ostwald ripening. Compared with other fruits, mandarin foams behave similarly to other acidic citrus (e.g., orange and lemon), where low pH facilitates protein unfolding, whereas neutral or high-sugar fruits (e.g., banana and mango) tend to have lower expansion due to less effective bubble stabilization.^81^

#### Determination and Validation of the Optimum Point

In the optimization study, the independent variables were selected as the egg albumen concentration in the range of 0–3% (w/w), carboxymethyl cellulose concentration in the range of 0–2% (w/w), and whipping time in the range of 3–7 min. Quadratic polynomial equations obtained from regression analysis were utilized to determine the optimum conditions for each response value (foam density, foam stability, and foam expansion). According to literature studies, conditions where foam density is minimized and foam stability and foam expansion are maximized are used to identify the optimum point.^82^ When the desirability function approach was applied, a total of 27 solutions were obtained for the optimum point. Table 8 presents the top six solutions that provide the optimum point. From the solutions determined by the program, the factor levels obtained in the first solution were selected as the optimal working conditions. According to the primary solution suggested by the desirability function approach, the factor levels are as follows: an egg albumen concentration of 3% (w/w), a carboxymethyl cellulose concentration of 2% (w/w), and a whipping time of 6 min and 20 s.

**Table 8:** First 6 Optimum Points Determined by the Desirability Function Approach

number	egg albumen (%w/w)	carboxymethyl cellulose (CMC) (%w/w)	whipping time (min)	FD (g/cm^3^)	FS (%)	FE (%)	desirability
1	3.00	2.00	6.20	0.258	348.048	273.293	0.858
2	3.00	2.00	6.16	0.259	347.836	273.197	0.858
3	3.00	2.00	6.28	0.258	347.833	272.683	0.857
4	2.98	2.00	5.48	0.258	347.092	273.192	0.857
5	2.04	2.00	3.05	0.258	346.219	274.152	0.857
6	2.02	2.00	3.08	0.258	346.168	247.179	0.857

To validate the optimum point results obtained through the desirability function, 3% (w/w) egg albumen and 2% (w/w) carboxymethyl cellulose were added to 100 g of mandarin juice, and the mixture was whipped for 6 min and 20 s. Foam production for the mandarin juice powder was carried out at the selected point. The results of the three validation experiments and the predicted values from the model are presented in Table 9.

**Table 9:** Optimum Point Verification Results

number	egg albumen (%w/w)	carboxymethyl cellulose (CMC) (%w/w)	whipping time (min)	FD (g/cm^3^)	FS (%)	FE (%)
1	3.00	2.00	6.20	0.252	362.962	288.888
2	3.00	2.00	6.20	0.275	365.384	303.846
3	3.00	2.00	6.20	0.258	318.518	314.814
average				0.262	348.955	302.516
predicted model				0.258	348.048	273.293

For each response parameter value, the statistical difference between the findings obtained from the validation experiments conducted at the optimum point and the values predicted by the model was determined by using a one-sample *t-*test in the SPSS software package. The one-sample *t*-test results for the response values are presented in Table 10. A comparison was made between the mean experimental values and the predicted values.

**Table 10:** Comparison of Average Experimental Values at the Optimum Point with Values Predicted from the Model

responses	predicted value	experimental value^a^	SH^b^	difference	%error^c^	*p*-value
FD (g/cm^3^)	0.258	0.262 ± 0.011	0.006	0.003	1.395	0.647
FS (%)	348.048	348.955 ± 26.386	15.234	0.907	0.260	0.958
FE (%)	273.293	302.516 ± 13.014	7.513	29.223	9.660	0.060

a Experimental results are given along with standard deviation.

b Mean standard error.

c % Error = (|y_den_ – yt_ah_|/y_den_) × 100.

According to the analysis results of the mandarin juice foam obtained from the validation experiments, all response values were found to be very close to the predicted values from the model, and the differences were statistically insignificant (*p* > 0.05). On the basis of these findings, the optimal foam production conditions were determined as 3% (w/w) egg albumen concentration, 2% (w/w) carboxymethyl cellulose concentration, and a whipping time of 6 min and 20 s. In the subsequent stages of the study involving the drying process, production was carried out using the findings obtained through the desirability function approach.

### Drying Process

#### Effects of Drying Process Conditions on Drying Time

It was determined that the total drying time varied significantly according to the drying method and drying temperature. It was determined that the drying times varied between 70.00 ± 0.00 and 180.00 ± 0.00 min in all drying process conditions (Table 11). The shortest drying time belonged to the drying process carried out at 70 °C using the hot air drying method. Figure 3 shows the visual of mandarin juice foams dried with hot air. The drying process using the freeze-drying method took longer than all drying processes using the hot air drying method. It was determined that the longest drying time belonged to the freeze-drying foam drying process carried out at −52 °C.

**Table 11:** Drying Time Values for Different Drying Methods in the Production of Mandarin Juice Powder

drying parameters	drying time (min)
TD 50	105.00[Table-fn t11fn1] ± 0.00
TD 60	82.00[Table-fn t11fn1] ± 2.35
TD 70	70.00[Table-fn t11fn1] ± 0.00
L −52	180.00[Table-fn t11fn1] ± 0.00

a–d The different lettering within the same column indicates that the various drying process conditions have statistically significant effects on drying time: *p* < 0.05. The lettering is arranged alphabetically from the largest to the smallest value. TD 50: tray dryer at 50 °C, TD 60: tray dryer at 60 °C, TD 70: tray dryer at 70 °C, and L −52: lyophilizer at −52 °C.

**Figure 3:**
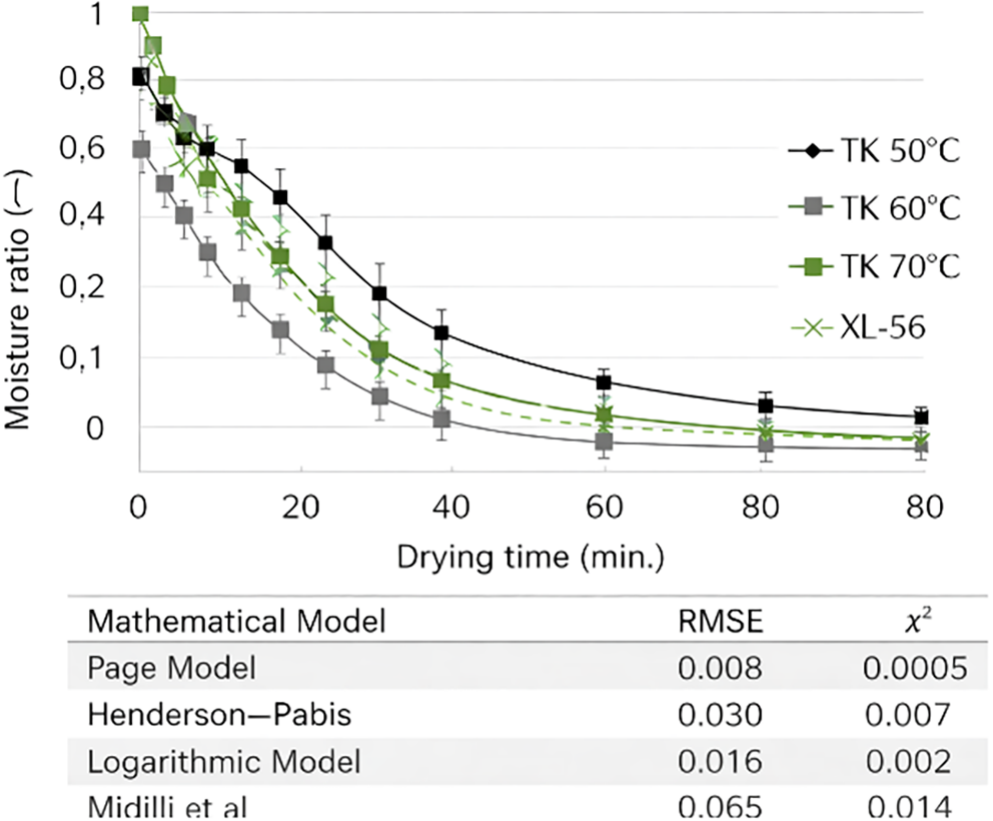
Moisture ratio (MR) of foam dried by hot air and freeze-drying methods, depending on drying time.

#### Effect of Drying Process Conditions on Drying Characteristics

In hot air drying processes (50, 60, and 70 °C), the initial moisture contents of the mandarin juice foams were determined as 82.41 ± 0.17%, 82.32 ± 0.08%, and 84.17 ± 0.23%, respectively; and in freeze-drying processes, the initial moisture contents of the mandarin juice foams were determined as 82.95 ± 0.83%. The difference between the initial moisture content values of the samples dried with different drying techniques was found to be insignificant (*p* > 0.05). Table 12 shows the initial moisture and initial free moisture contents for all drying temperatures. Figure 2 shows the change in total and free moisture content with drying time.

**Table 12:** Initial Moisture and Initial Free Moisture Contents of Mandarin Juice Foams

sample	initial moisture (%)	initial free moisture contents (kg water/kg DM)
TD 50	84.17[Table-fn t12fn1] ± 0.23	5.32[Table-fn t12fn1] ± 0.08
TD 60	82.32[Table-fn t12fn1] ± 0.08	4.93[Table-fn t12fn1] ± 0.03
TD 70	82.41[Table-fn t12fn1] ± 0.17	4.98[Table-fn t12fn1] ± 0.05
L −52	82.95[Table-fn t12fn1] ± 0.83	1.72[Table-fn t12fn1] ± 0.14

a–c Different letters in the same column indicate that different drying process conditions have statistically different effects on the initial moisture content and free moisture content: *p* < 0.05. Lettering is done alphabetically from largest to smallest. TD 50: tray dryer at 50 °C, TD 60: tray dryer at 60 °C, TD 70: tray dryer at 70 °C, and L −52: lyophilizer at −52 °C.

The initial free moisture content of the samples dried at 50 °C was found to be 5.32 ± 0.08 kg water/kg DM, the initial free moisture content of the samples dried at 60 °C was found to be 4.93 ± 0.03 kg water/kg DM, the initial free moisture content of the samples dried at 70 °C was found to be 4.98 ± 0.05 kg water/kg DM, and the initial free moisture content of the samples dried at −52 °C was found to be 1.72 ± 0.14 kg water/kg DM. While the difference between the samples dried at 60 and 70 °C was insignificant (*p* > 0.05), the difference between the free moisture content values of the mandarin juice powders dried at other temperatures was statistically significant (*p* < 0.05). It was determined that the free moisture content of mandarin juice foams decreased rapidly up to 60 min under all drying conditions, and the decrease in free moisture content slowed down in the later stages. It has been stated in the literature that in the drying processes of foods, the free moisture content decreases in the early stages of drying, and this decrease slows down as time progresses.^83,84^

The moisture ratio (MR) data of the mandarin juice powder samples were integrated into the experimental models in order to mathematically express the effects of different drying conditions on drying properties. The moisture change (MR) of the mandarin juice powders obtained under different drying conditions, depending on the drying time, is shown in Figure 3.

#### Mathematical Modeling

Based on the drying kinetic findings obtained within the scope of the thesis study, a compatibility test process using a total of seven different mathematical models (Lewis, Page, Henderson and Pabis, logarithmic, binary, exponential binary, diffusion approach) was carried out. Nonlinear regression analysis was conducted for different processing temperature values based on *R* ^2^, RMSE, and χ^2^ values to test the models’ compatibility. The obtained results are presented in Table 13.

**Table 13:** Nonlinear Regression Analysis Results of Semi-Empirical Models in Foam Drying of Mandarin Juice Using Hot Air and Freeze-Drying Processes

temperature	model	*R* ^2^	RMSE	χ^2^
TD 50	Lewis	0.975 ± 0.00	0.048 ± 0.01	0.002 ± 0.00
	Page	0.994 ± 0.00	0.024 ± 0.00	0.001 ± 0.00
	Henderson and Pabis	0.979 ± 0.00	0.045 ± 0.00	0.002 ± 0.00
	logarithmic	0.994 ± 0.00	0.034 ± 0.01	0.001 ± 0.00
	binomial	0.979 ± 0.00	0.045 ± 0.00	0.002 ± 0.00
	exponential binomial	0.975 ± 0.00	0.048 ± 0.01	0.002 ± 0.00
	diffusion approach	0.984 ± 0.00	0.039 ± 0.01	0.002 ± 0.00
TD 60	Lewis	0.973 ± 0.00	0.053 ± 0.01	0.003 ± 0.00
	Page	0.996 ± 0.00	0.020 ± 0.00	0.000 ± 0.00
	Henderson and Pabis	0.978 ± 0.00	0.121 ± 0.10	0.026 ± 0.03
	logarithmic	0.993 ± 0.00	0.033 ± 0.00	0.001 ± 0.00
	binomial	0.978 ± 0.00	0.048 ± 0.00	0.002 ± 0.00
	exponential binomial	0.973 ± 0.00	0.053 ± 0.01	0.003 ± 0.00
	diffusion approach	0.988 ± 0.00	0.033 ± 0.00	0.001 ± 0.00
TD 70	Lewis	0.970 ± 0.01	0.057 ± 0.01	0.004 ± 0.00
	Page	0.997 ± 0.00	0.017 ± 0.00	0.000 ± 0.00
	Henderson and Pabis	0.976 ± 0.01	0.050 ± 0.01	0.003 ± 0.00
	logarithmic	0.991 ± 0.00	0.036 ± 0.01	0.001 ± 0.00
	binomial	0.976 ± 0.01	0.050 ± 0.01	0.003 ± 0.00
	exponential binomial	0.970 ± 0.01	0.057 ± 0.01	0.004 ± 0.00
	diffusion approach	0.986 ± 0.00	0.038 ± 0.01	0.001 ± 0.00
L −52	Lewis	0.999 ± 0.00	0.011 ± 0.00	0.000 ± 0.00
	Page	1.000 ± 0.00	0.005 ± 0.00	0.000 ± 0.00
	Henderson and Pabis	0.999 ± 0.00	0.011 ± 0.00	0.000 ± 0.00
	logarithmic	1.000 ± 0.00	0.009 ± 0.00	0.000 ± 0.00
	binomial	0.999 ± 0.00	0.011 ± 0.00	0.000 ± 0.00
	exponential binomial	0.999 ± 0.00	0.011 ± 0.00	0.000 ± 0.00
	diffusion approach	1.000 ± 0.00	0.007 ± 0.00	0.000 ± 0.00

There are some coefficients in 7 different model equations selected as a result of literature studies. The coefficient values of the models used were calculated by performing nonlinear regression analysis with the SPSS package program. Table 14 shows the mathematical model coefficients.

**Table 14:** Coefficient Values of Mathematical Models Used in Drying Mandarin Juice Foams

temperature	models	
TD 50	Lewis	*k* = 0.031
	Page	*k* = 0.010, *n* = 1.307
	Henderson and Pabis	*k* = 0.033, *a* = 1.058
	logarithmic	*k* = 0.024, *a* = 1.160, *c* = −0.121
	binomial	*a* = 0.527, *b* = 0.532, *k*_0_ = 0.033, *k*_1_ = 0.033
	exponential binomial	*k* = 0.031, *a* = 1.000
	diffusion approach	*k* = 0.035, *a* = 1.165, *b* = 2.871
TD 60	Lewis	*k* = 0.040
	Page	*k* = 0.011, *n* = 1.372
	Henderson and Pabis	*k* = 0.043, *a* = 1.069
	logarithmic	*k* = 0.032, *a* = 1.163, *c* = −0.112
	binomial	*a* = 0.537, *b* = 0.532, *k*_0_ = 0.043, *k*_1_ = 0.043
	exponential binomial	*k* = 0.040, *a* = 1.000
	diffusion approach	*k* = 0.042, *a* = 1.197, *b* = 3.091
TD 70	Lewis	*k* = 0.048
	Page	*k* = 0.013, *n* = 1,424
	Henderson and Pabis	*k* = 0.051, *a* = 1.076
	logarithmic	*k* = 0.039, *a* = 1.164, *c* = −0.104
	binomial	*a* = 0.536, *b* = 0.540, *k*_0_ = 0.051, *k*_1_ = 0.051
	exponential binomial	*k* = 0.048, *a* = 1.000
	diffusion approach	*k* = 0.044, *a* = 1.134, *b* = 1.340
L −52	Lewis	*k* = 0.035
	Page	*k* = 0.022, *n* = 1.122
	Henderson and Pabi	*k* = 0.035, *a* = 1.0003
	logarithmic	*k* = 0.034, *a* = 1.013, *c* = −0.011
	binomial	*a* = 0.610, *b* = 0.393, *k*_0_ = 0.035, *k*_1_ = 0.035
	exponential binomial	*k* = 0.035, *a* = 1.000
	diffusion approach	*k* = 0.032, *a* = 1.061, *b* = 0.345

In order to select the appropriate model, the *R* ^2^, root-mean-square error (RMSE), and χ2 values for each temperature value were examined. The model with the highest *R* ^2^ value and the lowest RMSE and χ2 values was selected as the most appropriate model. In this context, for all drying groups, it was determined that the model that showed high compatibility with all drying processes was the ″Page″ model (*p* < 0.05).

When different studies on mandarin fruit were examined,^85^ mandarin peel waste was subjected to infrared drying at three different temperatures (50, 60, and 70 °C) at a constant air flow rate (2 m/s). It was determined that the model that was compatible with the study among the different models used was the ″Page″ model for all three temperature values. Ref^86^ aimed at drying mandarin slices at different temperatures (55, 65, and 75 °C) in an oven with a constant air flow rate of 1.3 m/s and in a vacuum oven with a vacuum pressure of 60 mbar and at explaining the drying kinetics of mandarin slices with seven different models. As a result of the study, it was emphasized that the best defined model for both processes was the ″Page″ model. Ref^87^ aimed at investigating the effect of osmotic dehydration pretreatment and the effects of drying conditions on the final product properties in vacuum microwave drying of mandarin fruit. After osmotic dehydration, the samples were dried at 960 and 1280 W microwave power, and the findings during drying were recorded. It was emphasized that the best model selected to explain the drying kinetics of osmotically dehydrated mandarins among the models used is the “Page” model. Different from these examples, ref^88^ added 2.1% (w/w) soy protein isolate, 2.75% (w/w) glycerol monostearate, 1.75% (w/w) carboxymethyl cellulose, and 5.10% (w/w) sugar to mandarin juice. The obtained mandarin juice foam was spread in different thicknesses (2, 4, and 6 mm) and subjected to the drying process at 180, 360, 540, 720, and 900 W microwave powers. It was stated that the “Midilli” model, “Weibull Distribution” model, and “Jena & Das” model showed the best fit.. But, in another study, ref^89^ aimed to investigate the effects of gum arabic concentration used at different rates (0, 5, 10, and 15 (w/w)) in the foam drying process dried in a tray dryer at 55 °C of melon fruit on the drying kinetic properties. 1% (w/w) methyl cellulose was added to each formulation, and the mixture was whipped for 10 min. Three different empirical models were tested in the mathematical modeling process. It was stated that the “Page” model was the most suitable model to describe the foam drying process of melon puree among all of the models. Similarly, ref^90^ aimed at drying the foams produced by adding 1% (w/w) glycerol monostearate to pumpkin puree at 45 and 65 °C by convective and foam drying methods. As a result of the drying processes, it was determined that the model that was most compatible with the study was the ″Page″ model. Likely, in another study, ref^91^ found that ″Midilli″, ″Lewis″ and ″Page″ models were compatible for all three temperature values of 50, 60, and 70 °C of Malay apple. Ref^21^ aimed at investigating the foam properties of sugar apple and performing mathematical modeling of the foam drying process in the tray dryer at 60, 65, 70, and 75 °C. In the study, they added egg albumen in the concentration range of 0–20% (w/w) and methyl cellulose in the concentration range of 0–0.50% (w/w) to sugar apple pulp and whipped the mixture for different times (5, 10, 15, 20, and 25 min) to obtain foam. As a result of the optimization studies, they found that the foam with the desired properties contained 15% (w/w) egg albumen and 0.37% (w/w) methyl cellulose, and the appropriate whipping time was 17.32 min. It was examined that the most compatible model was the ″Hii, Law, andCloke″ model. Additionally, ref^92^ evaluated the foam drying kinetics of mango puree at different temperatures (50, 60, and 70 °C) and spreading thicknesses (0.5, 1.0, and 1.5 cm). In line with this study, the “Page” model was the model that showed the best fit with the experimental drying curve data and was the most compatible model for all operating conditions. Differently, ref^93^ carried out mathematical modeling of the foam drying method used to obtain powder product from guava fruit pulp. Two different egg albumen concentrations (4% (w/w) and 8% (w/w)) and three different temperatures (75, 80, and 85 °C) were used in the drying process. The compatibility of the “Lewis” model, “Page” model, “Midilli” model, and “Logarithmic” model was tested, and it was stated that all models were in high agreement with the experimental data, and the “Midilli” model was the most compatible model. Also, it was reported that the “Page” model was suitable for both foaming agent combinations used (3.5% (w/w) egg albumen +0.25% (w/w) carboxymethyl cellulose +0.25% (w/w) xanthan gum and 7% (w/w) egg albumen) for Uvaia pulp powder.^94^ Ref^22^ obtained lemon juice foam by adding two different foaming agents (15% (w/w) glycerol monostearate and 10.5% (w/w) egg albumen) to lemon juice mixed with sugar, salt, and honey and by beating the mixtures for 20 and 30 min, respectively. The obtained foams were spread in a 0.5 cm thickness and dried in a tray dryer at 60 °C, and the drying kinetics were investigated by mathematical modeling. It was determined that the “Henderson and Pabis” and “exponential binomial” models were the best models to describe the drying properties of lemon powder.

In the studies in the literature, it has been determined that the drying method and temperature, the speed of the drying air, the type and amount of foaming agent and foam stabilizers used in obtaining the foam to be dried, the foam spreading thickness, the capacity of the dryer system, etc., directly affect the drying kinetics. As a result, it has been determined that the mathematical models differ from each other, and different compatibilities are observed as a result of the modeling.

## Conclusion

The mandarin juice foams produced at the recommendation point determined by the desirability function approach were dried with hot air and freeze-drying techniques to determine the optimum drying time and drying characteristics. Optimum production conditions for mandarin juice foams with carboxymethyl cellulose were a whipping time of 6 min 20 s, an egg albumen concentration of 3% (w/w), and a carboxymethyl cellulose concentration of 2% (w/w). The drying times for temperatures of 50, 60, 70, and −52 °C were determined as 105 ± 0.00 min, 82 ± 2.35 min, 70 ± 0.00 min, and 180 ± 0.00 min, respectively. It was determined that the lowest drying time belonged to the drying process carried out at 70 °C using the hot air drying method. The ANOVA findings suggested that the generated models had a higher *R* ^2^ value (>85%), a small CV value, significant model F values (*p* ≤ 0.05), and nonsignificant ‘lack of fit’. Consequently, the regression model was assessed for fit to the experimental values and was determined to be well-fitted. The optimized parameters were verified, and there was good agreement between the experimental results. Considering all of the data, these findings indicated that optimization of the foam-mat drying process was critical, since the varying concentrations of EA, CMC, and whipping time affected foam characteristics. A total of 7 different thin-layer drying models of mandarin juice powder samples were analyzed, and it was determined that the compatible mathematical model was the ″Page″ model for different drying process conditions. It is thought that this study carried out in this context will benefit the scientific literature. In further studies, the usage of the foam-mat-dried powders in the food products could be investigated.
